# Sport, the arts, and fans’ loyalty: the role of color for sport fans

**DOI:** 10.3389/fpsyg.2023.1239085

**Published:** 2023-10-13

**Authors:** Ron Schleifer, Ilan Tamir

**Affiliations:** Moskowitz School of Communication, Ariel University, Ariel, Israel

**Keywords:** sport, art, fans, esthetic, colors

## Abstract

Sport fandom has attracted far-ranging research attention. Fans’ commitment, loyalty, and sense of affinity are abundantly expressed in a long list of life practices. Precisely in this context, the color that identifies each sports team has also become an important element of the game but also a crucial component of fans’ identities. The present article focuses on the esthetics of team uniform colors and identifies the artistic roles they fill. Among other things, the article addresses the important role of color, for example in identifying and distinguishing figures on the pitch from the background by creating clear borders between the elements on the pitch that compete for fans’ attention; in the extension of fans’ emotions through the connection between team and color; in creating the opportunity for spectators to participate fully in creating the esthetic experience on the field through the intensive use of team colors; and in enhancing a team’s halo effect through the association of the team with its colors. On a deeper level, the article states that in an era of commercialized sports, the team color remains the clear and consistent symbol of the team that preserves the identity of the fans.

## Theoretical background

### Esthetics and color

Esthetic experiences have a profound effect on our lives and affect decisions and behavior, far beyond what is generally called art (Brielmann and Pelli, 2018). Schweid (2004) contends that esthetic emotion is a tool in the service of evolution and that the esthetic experience contributes to man’s well-being. Research has proved that esthetics affects our mental health, the healing process in patients (Ulrich, 2001 Evans, 2003), cognitive function in general (Wells, 2000), and even the performance of more prolonged sports activity such as walking (Ball et al., 2001).

Esthetic emotion has several origins, and while the sense of sight may be central, it is not the only source. An esthetic experience is obtained from the activation of several senses simultaneously—in the case of sports, by both the players and the spectators. Thus, the effects of contact and friction on the field and in the bleachers, the sense of smell in the stadiums (food, sweat, smoke), the sense of hearing which is considered to be of great importance at sports events, etc. can be objects of research. In the present study, we focus on the esthetic experience deriving from the sense of sight, due to its dramatic contribution to esthetic emotion in general and in sports in particular. Visual esthetics has indeed attracted extensive research attention, but, strangely enough, the role of color, considered an important esthetic component, has remains somewhat in the shadows (Nascimento et al., 2021).

The role of color in our lives is far greater and more important than might be thought. It is present in almost every aspect of our lives, affects our beliefs and feelings, and the decisions we make, and is even in our dreams (Elliot and Maier, 2014). Color is considered one of the most important visual components in our lives and even the most powerful information channel of all human senses (Suk and Irtel, 2010). Vecera et al. (2004) stressed the ability of color to create distinctions between figures and their background and between static objects and objects in motion, and so helps to identify a person or an object in a space. This is of genuine importance in the dynamic and crowded sports space.

### Sport and society

Sport has become one of the most important and popular institutions in modern society. It enthralls billions of people worldwide and bridges over geographic, cultural, ethnic, and other differences (Kennedy and Hills, 2015). The popularity of sports is enormous, and its fans are exceptionally loyal and committed to the team and its players (Wann and James, 2018). It is no coincidence that many compare sports to religion, based on their many material and symbolic commonalities (Bain-Selbo and Sapp, 2016).

One of the main reasons for sports’ unprecedented popularity and consequently the strong dedication of its fans, is the fact that for the fans, sport represents something far greater than the game itself. A sports team represents profound elements of fans’ identities. As a result, most sport events are heavily charged with historic, political, social, economic, and other meanings, far beyond the game itself (Tamir, 2022). It is therefore no surprise that the audience’s involvement and commitment is exceptional in comparison with other social and popular arenas.

As a result of this, we can see a particularly impressive commitment of sports fans to their teams, as expressed in constant sacrifice—financial, physical, emotional, and many other practices (Levental et al., 2021) that emphasize the deep connection between the fans and their team. The other side of the coin highlights the less beautiful sides of the sport (Galily et al., 2016), including the violence (Levental and Ben-Eli, 2022) that comes from exactly same places.

Sports in general and football in particular are full of emotionally charged moments (Ng and Kidder, 2010), which allow activists and spectators to discharge emotions (Elias and Dunning, 1986).

Elias and Dunning in the Quest for Excitement in Leisure (Elias and Dunning, 2008) pointed to the sport’s distinctive essence. The sport, according to them, is based on a deliberate build-up of tensions over a period of time, designed to culminate in a climactic release, without which it would cease to be exciting or enjoyable. That is, the pleasure is derived from “controlled decontrolling of emotional controls.” Accordingly, the sport provides its players and fans with a wide and extreme range of emotions and allows the expression of emotional outbursts not acceptable in other areas of daily life.

In addition, in the modern cultural age, sports events may represent almost single opportunity for fans’ to challenge their identities and examine them against a competing identity. On the basis of this notion, the author George Orwell offered an accurate description of sports events as “war minus the shooting” (Orwell, 1945). It is therefore unsurprising that over the years, sports has adopted militaristic rhetoric (Tamir et al., 2017).

Moreover, it should be remembered that due to its competitive nature, sport is also a rare opportunity for people to challenge physical limitations and the forces of nature. The innate ambition of sports to break records drives sportsmen and women to run faster, to jump higher, and to throw stronger. Therefore, watching a human body at its peak is in itself an impressive esthetic experience and is also considered one of the secrets of sports’ magic and appeal (Mumford, 2013).

Like national symbols, sport teams, which as we have already said are perceived as extensions of identity (national and others), are identified with symbols and colors. Every sport team is identified with the color of their uniforms, which their fans also adopt in their attire and accessories. Team colors have long been a part of fans’ identity, irrespective of a specific game (Tamir, 2021). Although colors have gained key status in the sporting arena, color has received scant attention in sports research. Research on the subject has studied the effects of color on sporting achievements or on referees’ decision making (Hagemann et al., 2008; Krenn, 2014) but has rarely investigated color in an esthetic context. The present research focuses on the artistic and esthetic role of color on the sports fields, especially for sport fans, which has become of greatest importance in the sporting arena.

## Discussion—the esthetics of color in sport

Sports has a unique esthetic that derives from its internal logic (Mumford, 2019). The main purpose of sport is obviously the competition, but at the same time it creates an esthetic value.

The esthetic structure of sports is largely fixed—infrastructure, rules, and other basic principles. Even the players’ position on the field is structured and constrained in many fields of sport, but the harmony between the players in team sports, the superhuman effort of sportsmen and women who pushing their physical limits, and obviously the spectators’ presence in the stadiums, are all part of the esthetics of a sports event. The massive media presence at sports events makes the unique esthetic elements accessible and observable.

Precisely in this context, a team’s colors play several important esthetic functions in sports: distinguishing a figure from the background and creating boundaries between the competitors, extending the duration of esthetic emotion, and creating an opportunity for the audience to participate fully in shaping the game’s esthetic experience.

### Identifying figures and distinguishing them from the background

Competitive sports between individuals or teams, in a limited pre-defined space creates a dynamic and constantly changing environment. In the spirit of the ethos of modern sports—“faster, higher, stronger”—sports activities themselves are conducted at an extremely high intensity and pace. The ability to mark the competing objects on the field is of enormous importance for the experience of the game and watching it, and become an extremely challenging task in such an environment, certainly when considering the fact that more than 20 athletes are engaged on the field simultaneously in some fields of sport, in addition to referees, trainers, and players on the bench (substitutes). The size of the stadiums and the massive and active audience within them also make the task of identification difficult. Since some branches of sport are played in the open air, and the weather conditions (e.g., mist, rain, wind), add to the complexity of the task by limiting one’s vision and making it difficult to identify players on the field (Goldschmied et al., 2020). Color therefore plays an extremely important role in the identification process by creating the essential contrast between team members and their opponents.

Much sports takes place in open spaces (which are characterized by a large number of stimuli and concurrent dynamic developments) in which the players, the referees, and the spectators scour the area to identify threats and opportunities. Identifying players, in this case by the color of their uniforms, is therefore critical. The open area demands players and spectators to attend to multiple dimensions simultaneously, hence the great importance of color and its prominence—in helping to survey the situation on the field, in order to build up a current picture of the situation and make good decisions. In this context, too, color plays a dramatic role, since it allows for information to be processed by players and spectators, for the speed of the figures to be identified even without processing the finer details of each image. In a sports event, we are in fact completing the information with the help of the esthetics of color.

Colors are also helpful to the sportsmen and women themselves who are in constant motion, to the referees, and as previously stated, to the spectators. In esthetic terms, color plays a part in identifying figures and distinguishing them from the background, due to the creation of clear boundaries between the competing elements on the field. For this reason, for example, the goalkeepers of soccer teams wear a different color of shirt from the other players, so as to mark them as having unique status (they may handle the ball), similar to libero’s role in volleyball, to whom different rules apply from the other team members. This is also true of the color of the ball in many branches of sport, allowing it to be identified at any given moment and to be distinguished from the other objects on the field and so too for the color of the referees’ shirts. On the same grounds it is also possible to argue that the color of the surface on which the competition takes place, is of importance and plays a special role. Thus, for example, the green color of the grass on which many branches of sports are played, makes it a space that maintains the games’ true origins as a spontaneous neighborhood game before the sport was disciplined through defined economic-professional codes.

The intensive entry of the media into sports events, given the enormous investment in purchasing broadcasting rights (Tamir and Lehman-Wilzig, 2023), has accelerated the need for clear distinctions between the forces operating on the field. Televised sports broadcasts give viewers high-quality and unique viewing angles, as a result of the many resources (multiple quality cameras, advanced technology, etc.) allocated to the event. In some branches of sports, games are filmed to input referees’ decisions (that are sometimes made at an accuracy of centimeters) and so, for one reason or another, the image of what is happening on the field must be as clear and incontrovertible as possible.

The categorical distinction and identification afforded by the colors of the shirts nonetheless exacts a toll. Research has shown that color *per se* affects refereeing and assessments of the athletes in a variety of branches of sport, due to the fact that some colors attract more attention than others or are associated with certain cultural phenomena, such as aggressiveness (Hill and Barton, 2005; Attrill et al., 2008; Ilie et al., 2008).

### Extending the duration of esthetic emotions

The debate over “imagined communities,” which are associated with the cultivation and reinforcement of national sentiments (Anderson, 2006) was warmly adopted by sports researchers who sought to describe the solidarity and comradeship of sport fans around their team (Kalman-Lamb, 2021). Both in the national context and the sports context, a member of the community needs clear systems of symbols to help him feel attachment to the community and connect with its values. In fact, colors become the symbol that is most strongly identified with a sports team.

The initiation of young fans, largely by the father of the family (Tamir, 2022) includes the adoption and acquisition of the esthetic emotion. To bring their children into the circle of the team’s supporters, parents use various methods, which include emphasis on identification with the team’s color, and no less important, antagonism to the opposing team’s color. This is generally expressed in attire, bedroom decorations, and accessories. Consequently, purchase of the club’s shirts, scarves, and other branded merchandise (Derbaix and Decrop, 2011) plays an important part in the inculcation of the esthetic emotion in each new generation of fans.

The commodification of sports allows fans to purchase club-related merchandise—and perhaps even exploited their fierce attachment to the club—to reinforce their identification as fans and their attachment to the club and to the community (Vamplew, 2018). To a great extent, the team’s color can be seen as adorning all the merchandise, as a kind of extension of the fandom and the emotion that accompanies it. Since a team’s games are played approximately once a week and are seasonal in nature with long breaks, a team’s colors also allow fans to extend the duration the esthetic emotion they associate with their teams well beyond the actual game.

It is important to stress that the extension of fandom through color is used both internally (the fan and his/her feeling toward the team) and externally (the community and its relationship to the fan). In effect, this notion is consistent with the “halo effect” (Kahneman, 2011). The argument is that one’s initial general impression or the impression of a salient feature biases one’s thinking and influences the way in which we judge other qualities. We are inclined to apply the qualities and feelings we have for a product, or in our case a team, to other elements related to the team’s fans. In the sporting context, Hickman and Lawrence (2010) showed that fans are inclined to give higher ratings to issues related to their favorite team and give lower ratings to those associated with their team’s rivals. In the same vein, it could be argued that a prominent color on and off the field enfolds the full set of qualities that fans associate with the team and its community of fans. This implies that I will begin to attribute positive qualities to a player, just became he is wearing “my team’s” shirt, even if I did not like him a short time ago when he played for the opposing team. This is also the case with regard to fans. When a person whom I do not know at all walks along the street with a shirt in my team’s colors. I will probably associate him with the attributes and feelings I have toward the team itself. Colors endow fans with social status and affirms their membership in the community.

It should be emphasized that this argument is based on the notion that one’s esthetic emotion develops over time, meaning that esthetic taste is acquired over a lifetime. Bourdieu (1978) argues that the favoring of different types of sports are derived from the luxury of having spare time, public image and norms. He refers to such elements as “ethical or esthetic accomplishment which are or seem to be contained in each sport.”

Since sports fandom is, to a large degree, “inherited,” early family experiences probably affect young family members’ appreciation of specific colors, and the emotions associate with them.

### Communities as active partners in creating esthetic emotion in sports

Spectators are considered an important element of the game, to the extent that they are occasionally termed “the 12th player” (in addition to the 11 players in every soccer team). Researchers have found that spectators (or their absence) have a genuine impact on what happens on the field. Thus, for example, spectators were found to have a direct, psychological, and physiological effect on the players—who, for example, experience hormonal changes (in the level of testosterone) in the games, depending on the pressure exerted by the audience (Carré et al., 2006; Fothergill et al., 2017); on referees and their decisions in the game (Anderson et al., 2012). Studies also found that during the Covid-19 pandemic, games that were played without an audience typically involved fewer emotional situations, and differences could be observed in the number of fouls and goalmouth poaching (Leitner and Richlan, 2021).

The affinity between spectators and the team, as previously stated, is important at almost any time and place, but the games in the stadiums are an important intersection of a team, its fans, and the fans of the rival team. It is therefore a good opportunity for fans of both teams to prominently externalize their feelings for their team, for better or worse, in chants and expressions of protest, which are also directed at the rival team (Schoonderwoerd, 2011; Tamir, 2020). In any event, audiences become full partners in the creation of the esthetic experience on the field, through the intensive use of the team’s colors—from attire, through banners and flags, to entire pyrotechnical routines. Color gives audiences an opportunity to become full partners in the creation of the esthetic experience on the field.

In other word, color is used on the field to create an esthetic of beauty by the audience who are wearing shirts and scarves in the team’s colors. Many spectators also use the bleachers for impressive and extremely creative displays of esthetic enjoyment.

## Conclusion

Color plays a central role in our lives, on conscious and unconscious levels. It affects the way we see reality and make judgments and decisions. In the world of sports, color is essential, yet its importance extends far beyond the symbolic. Color serves as a key symbol in the identification of groups. The basic and central objective of color is to differentiate between the competitors on the field, but it also fills esthetic roles, as presented in this article. Thus, for example, color allows the creation of clear boundaries between the players and clearly marks out figures and tasks on the field. Moreover, color is also what makes the audience on the field a partner in creating a game’s esthetic experience.

Televised sports broadcasts have enhanced the role of color and even elevated its use. Fans worldwide have begun to display their affection for the club with products in the club’s colors and to echo this in their social media. In other words, color has become an esthetic agent of the highest order in the world of sports.

In practice, modern sports teams can be likened to the Ship of Theseus allegory. The philosophical question at the heart of the story is whether a ship with all its parts replaced remains the same ship? By the same token, in an era of commercialized sports, players and coaches move from team to team so frequently, and the essence of the team is seemingly emptied of its content. And in this situation, the identity of the team is actually derived from the tradition and especially from its symbols. The color, which became the most distinct and recognizable symbol of the sports teams, therefore became a torch that carries with it a significant value charge and a complete heritage of identity.

## Author contributions

This work is an equal shared effort paper. RS has contributed the art perspective and IT the sports perspective. The combined work produced a novel and unusual combination between two subjects that normally are not associated together. All authors contributed to the article and approved the submitted version.
